# 

**Published:** 1889-08-10

**Authors:** 


No. 150, Vol. VI.—August 10th, 1889.
Registered at the General Post Office as a Newspaper.]

Monthly Parts, 6d.; post free 7½d.

Ditto per Annum, 7s. 6d., post free.

